# Event and Comment

**Published:** 1912-08-15

**Authors:** 


THE
DENTAL REGISTER.

Vol. LXVI. August 15, 1912. No. 8

### EVENT AND COMMENT.

NERVE SUPPLY OF THE DENTINE. We are giving considerable space in this issue to a reprint of Dr. Mummery's recent contribution on the distribution of the nerves of the tooth pulp. This is the most painstaking and in some respects the most conclusive work ever done on this important subject. If Dr. Mummery's findings are established by future researches a much discussed problem of the most practical value will be settled and our methods of handling pathologic conditions of the dentine will be placed on a scientific basis. We have learned to handle sensitive dentine fairly well, but we shall now become intelligent as to means used in its treatment.

CLEANING TEETH. We protest this title but for our purpose nothing else will serve to make this comment. The May issue of the *Dental Dispensary Record* contains the statistics of the treatments given in the three Rochester Dispensaries for the months of March and April, and we note with regret that less than one in ten of the treatments are recorded under the heading of "cleaning teeth." We have noticed a similar proportion in the statistics elsewhere published and we can not refrain from commenting on what we consider a most important oversight in the conduct of these clinics. There can be little doubt that every one of the 1332 patients treated in the Rochester clinic during the months of March and April needed to have their teeth cleaned professionally, and what is more important they should have been given a thorough treatment and such advice as should have made them intelligent on the subject of the care of their own teeth. It would seem that nine tenths of the time and energy of the dentists in charge of these clinics had been expended in relieving the pain of diseased teeth and repairing the loss of tooth substance from caries. We do not know how much instruction was given in connection with these operations, and we can probably assume that only the usual amount of real instruction accompanied the actual cleaning operations. We are probably justified in saying that the amount of real hygienic instruction given in these clinics is far short of what it should be to justify the expense of money and sacrifice of time put into it by busy professional men. Of course actual repair and relief operations must be made, but the chief business of these clinics ought to be to impart instruction that will help these children to properly value their teeth and compel them to give them some measure of attention in the way of a systematic mouth toilet. We realize that this instruction can not be wholly didactic, the theory of oral sanitation can be imparted most impressively by an actual demonstration on the individual, not as an atonement for neglect, but a thorough prophylaxis treatment in which the patient become so interested because of the benefits and comforsts resulting that he will not easily forget the lesson, and will want to return again and again for similar treatments is what counts for future prophylaxis.

